# Bacterial colony size growth estimation by deep learning

**DOI:** 10.1186/s12866-023-03053-y

**Published:** 2023-10-26

**Authors:** Sára Ágnes Nagy, László Makrai, István Csabai, Dóra Tőzsér, Géza Szita, Norbert Solymosi

**Affiliations:** 1 Centre for Bioinformatics, University of Veterinary Medicine, 1078 Budapest, Hungary; 2 Autovakcina Ltd, 1171 Budapest, Hungary; 3 Department of Physics of Complex Systems, Eötvös Loránd University, 1117 Budapest, Hungary

**Keywords:** Bacterial growth rate, Neural network, Deep learning

## Abstract

The bacterial growth rate is important for pathogenicity and food safety. Therefore, the study of bacterial growth rate over time can provide important data from a medical and veterinary point of view. We trained convolutional neural networks (CNNs) on manually annotated solid medium cultures to detect bacterial colonies as accurately as possible. Predictions of bacterial colony size and growth rate were estimated from image sequences of independent *Staphylococcus aureus* cultures using trained CNNs. A simple linear model for control cultures with less than 150 colonies estimated that the mean growth rate was 60.3 $\mu m/h$ for the first 24 h. Analyzing with a mixed effect model that also takes into account the effect of culture, smaller values of change in colony size were obtained (control: 51.0 $\mu m/h$, rifampicin pretreated: 36.5$\mu m/h$). An increase in the number of neighboring colonies clearly reduces the colony growth rate in the control group but less typically in the rifampicin-pretreated group. Based on our results, CNN-based bacterial colony detection and the subsequent analysis of bacterial colony growth dynamics might become an accurate and efficient tool for bacteriological work and research.

## Introduction

Bacteria reproduce by simple division, the rate of which is fundamentally influenced by the environment and the characteristics of the bacterium. The rate of bacterial multiplication is important for pathogenicity [1, 2] and food safety [3]. Therefore, the study of bacterial growth (multiplication rate) per unit of time can provide important data from a medical and veterinary point of view. Several developments for the automated monitoring of the growth rate exist. In liquid cultures, the quantification of turbidity (optical density), electrical conductivity, or redox potential can be used for this purpose [4, 5]. As a result of these methods, data can be derived on the growth characteristics of the entire culture. However, we do not obtain information on the growing differences of individual bacteria, colony-forming units, which may be important in certain cases (e.g. persister cells) [6]. When bacteria are cultured on solid media, the growth rate is estimated from the change in bacterial colony size, which also allows differences in the growth characteristics of the colony-forming units to be studied. The most common solutions [7–11] involve digital image analysis, used to detect colonies and then measure their size relying on a threshold-based approach. A limitation of these approaches is that objects in the image that are not colonies (e.g., pieces of the wall of a Petri dish, air bubbles) may also appear in the result as colonies [12]. More recently, the potential of laser speckle imaging (LSI) for the study of bacterial colony growth has been demonstrated by Balmages et al. [13]. This approach allows the identification of pixels in images of cultures that do not change over time and those that do, using the laser as a coherent light source. It can therefore be used for the study of changes at the edges of colonies, and thus their growth. Although LSI can be used to overcome these shortcomings of the threshold-based approach, its application is currently more challenging.

In our work, we investigated the applicability of convolutional neural networks (CNNs) for estimating growth rates from time series of digital images of bacterial cultures. For the detection of bacterial colonies, these algorithms are promising tools to address the above-mentioned weaknesses of threshold-based approaches [14, 15]. If the detection of bacterial colonies is done to count the number of colonies, then the main goal of training CNN is to find the colonies. Less important is how well the dimensions of the predicted object match the dimensions of the colony. On the contrary, if we want to study growth rates, we need to be able to measure the size of the detected colonies as accurately as possible. This also means that we need to perform model selection according to such predictive measures of neural networks in order to obtain the most accurate outputs. With these considerations in mind, we trained and selected CNNs for the estimation of colony sizes and, as a result, growth rates.

## Methods

To detect bacterial colonies in the Detectron2 [16] environment, 10 pre-trained Faster R-CNN [17] models (R_50_C4_1x, R_50_C4_C4_3x, R_50_DC5_1x, R_50_DC5_3x, R_50_FPN_1x, R_50_FPN_3x, R_101_C4_C4_3x, R_101_DC5_3x, R_101_FPN_3x, X_101_32x8d_FPN_3x) were trained. For this purpose, our research group has previously created a manually annotated dataset (with bounding boxes enclosing the colonies) [12]. This dataset contains digital records of 24 bacterial species, 369 cultures with 56,865 annotated colonies. The dataset was randomly divided into two-thirds and one-third training and validation sets. Since the images were of different sizes, they were transformed to a uniform size ($6200\times 6200$ pixels) for training. Each pre-trained model was trained (base learning rate: 0.001, batch size per image: 10240) through 100 epochs and validated after every 100 iterations. During validation, we always recorded weights with a smaller validation loss compared to the previous smallest weights. Thus, the training resulted in a collection of the best weights for each of the 10 pre-trained models.

We performed bacterial colony detection predictions using the best weights and an independent image collection. The dataset used to investigate bacterial colony growth consisted of the unannotated digital images generated and shared on Figshare by Bärr et al. [11]. The authors took digital images of 22 *Staphylococcus aureus* cultures in every 10 min. From each culture, 410 or 423 recordings were made. Of the 22 cultures, 8 were control (without any antmicrobial treatment, Ctrl), and 14 were pretreated with rifampicin antibiotic (Rifa) for 24 h immediately prior to culturing (Table 1/A). Based on best weights, bacterial detection prediction was performed for images numbered 1-410. For the 410 records of 22 cultures (transformed to $6200\times 6200$ pixels), predictions (bounding box coordinates, object prediction probability) obtained with each model were stored. CNN training and predictions were performed on a Tesla V100 32GB GPU.

Further processing of the data and plotting of the results were done in R-environment (v4.2.1) [18] using the packages broom [19], broom.mixed [20], ggplot2 [21], sf [22], tmap [23], and xtable [24]. The bounding boxes around the colonies that were predicted with each model’s best weights were later filtered; only those with object prediction probabilities above 0.5 were retained for further analyses. Simple feature polygons were generated from their coordinates. From the predicted objects at 410 recording time, the ones with an object prediction probability greater than 0.95 were extracted. These represented the final state and size of the colonies.

Based on our experience, we assumed that in the case of *S. aureus*, the colonies’ center does not shift significantly during growth. Therefore, when tracking the growth of colonies, we assumed that the bounding box describing the latest state of the colony mostly contains the area determined during the previous predictions. Accordingly, for each bounding box describing the final colony state, we extracted the bounding boxes predicted at the previous time points that fell completely within the final one. In order to compare our results with the work of Bärr et al. [11], we used the radius of the colonies to describe their size. We estimated the colony radius by taking half of the width of the predicted bounding boxes. As the dataset reported by Bärr et al. [11] is not an annotated one, we were unable to use traditional metrics of prediction quality in model selection, so we selected the best model based on the following considerations. Despite the fact that the centre of the S. aureus colony does not shift significantly during culturing, there were bounding boxes that did not fit perfectly into the bounding box describing the final state of the colony. These outlying bounding boxes were omitted from the time series associated with the colonies, so that there were time series with fewer and those with more bounding boxes. The more data points we can use, the more accurate the growth rate estimate. In addition, in terms of the coherence of the model’s prediction, we would expect it to predict as many elements of the time series as possible that fall within the final bounding box for a given colony. Therefore, of the models tested, the one that contained the largest number of bounding boxes for each colony was considered the best. However, visual inspection of the predicted bounding boxes also showed that some models had systematically larger distances between the edges of their boxes and the boundaries of the colonies, while others had smaller distances. Since the boxes that provide a more accurate estimate of colony size are those with smaller distances between their bounding edges and the colony contour, we considered the best models to be those that consistently estimated smaller colony sizes. The median length of the bounding box series, the mean and standard deviation of the bounding box width were calculated for the time series of each bounding box for each of the models examined. We selected the model as the best one with the largest median length of the series of bounding boxes and the model with the minimum mean and standard deviation of the box widths. The dimensions of the bounding boxes predicted by this model were used to estimate the growth rates shown.

Following the work of Bärr et al. [11] we used the colony growth rates predicted during the first 24 h of culturing. Bärr et al. [11] estimated growth rates per culture using a linear model with colony size as the dependent variable and incubation time as the explanatory variable. The regression coefficients obtained per culture were averaged over the cultures of the Ctrl group with less than 150 colonies. For comparability, we used the same approach to estimate the growth rate. However, this approach assumes that the cultures are perfectly identical in all respects and that each measurement is independent. In reality, repeated measurements on the same cultures are not independent of each other and cultures will inevitably differ somewhat (e.g. in number of colonies, placement in the thermostat or thickness of the culture medium). In such cases, we can expect more extensible and robust results if we use a model that takes these aspects into account. Therefore, in addition to the above approach, we used a mixed effects linear model for growth rate estimation where colony size was modelled as the dependent variable with incubation time as a fixed factor and culture as a random factor.

The growth rate of bacterial colonies is influenced by how densely they are distributed in the culture. The number of other colonies in the surroundings of each colony that can affect its growth was characterized by the number of close neighbors (NB). We considered two colonies as close neighbors if the bounding boxes between them overlapped to any extent.

The time of the first colony appearance was estimated as follows. The age of each culture was assigned to the bounding box of each colony of each culture. These were pooled into a time series per culture. The minimum of these time series per culture gave the time of the first colony appearance.

## Results

**Fig. 1:**
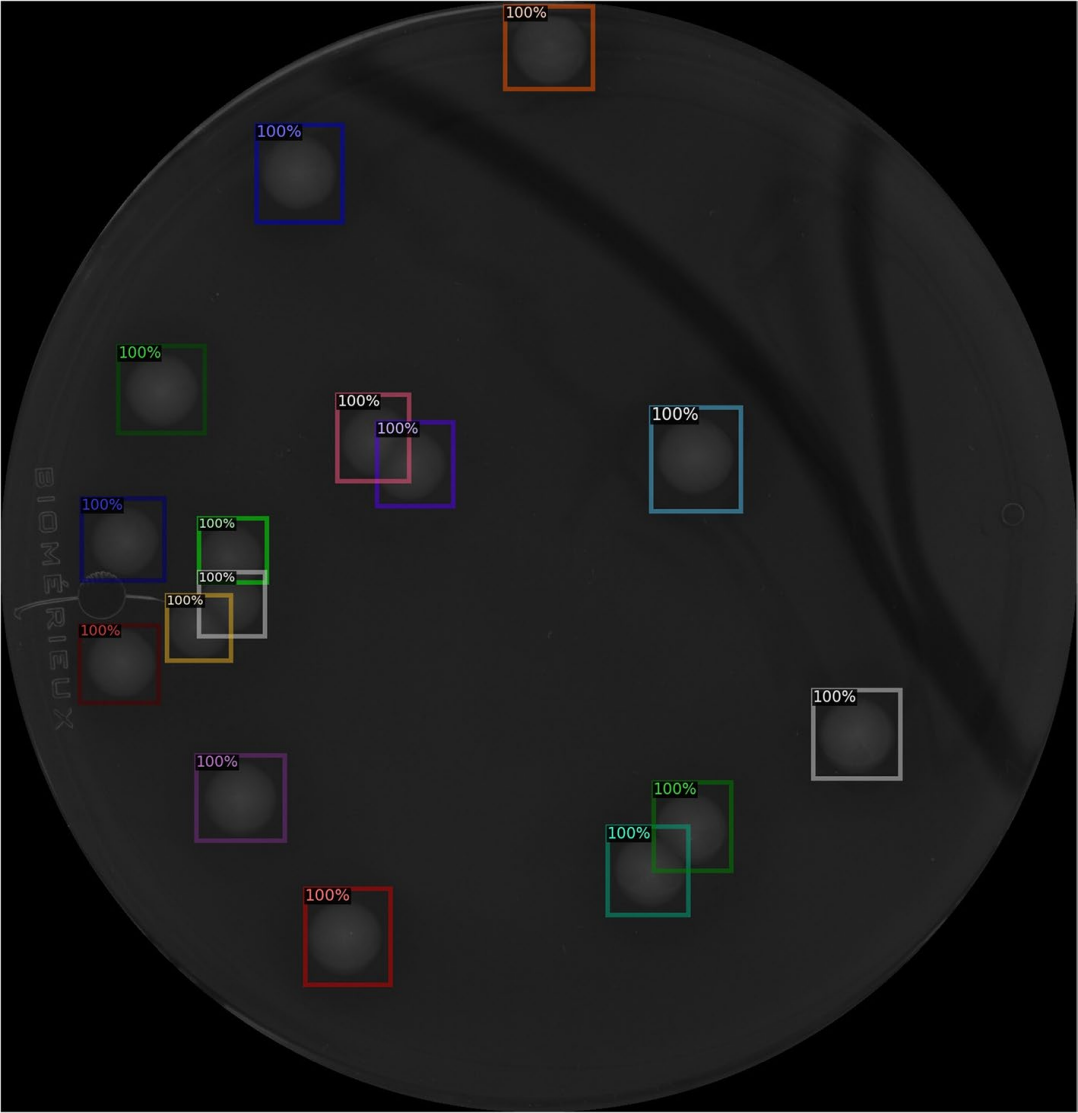
Bounding boxes on an image of Ctrl_1 culture predicted by the neural network. The colors have no meaning, they only aid separation in the case of overlapping boxes. The percentages indicate the confidence the algorithm assigned to the detected object

For the pre-trained model X_101_32x8d_FPN_3x, we obtained the largest median length ($n=323$) for the set of bounding boxes and the smallest mean (3.39 mm) and standard deviation (1.32 mm) for the width of the boxes. Regardless of the number of colonies, the trained neural network processed a single culture record in 0.31 seconds. This time included converting the image to a uniform size, predicting the bounding box of the colonies, and tabulating the data describing them. The bounding boxes on an arbitrary image of the Ctrl_1 culture are shown in Fig. 1.

**Fig. 2:**
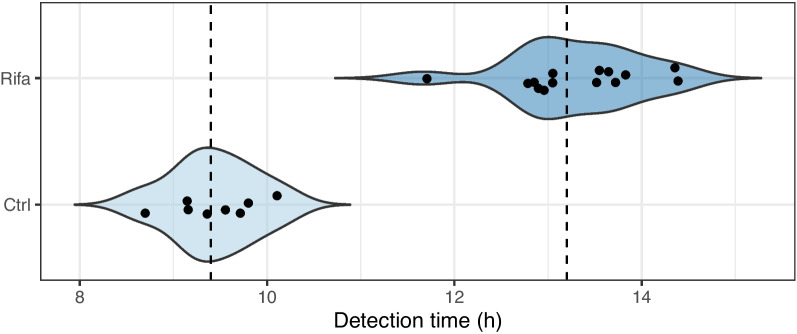
The time until the detection of the first colony in each culture in the two groups. Dashed lines indicate the median of the groups

Figure 2 shows the time when the first colonies were detected in each culture. While the median time to first colony detection in the Ctrl group was 9.4 h, the median time to first colony detection in the Rifa group was 13.2 h, with a difference of 3.8 h.

**Fig. 3:**
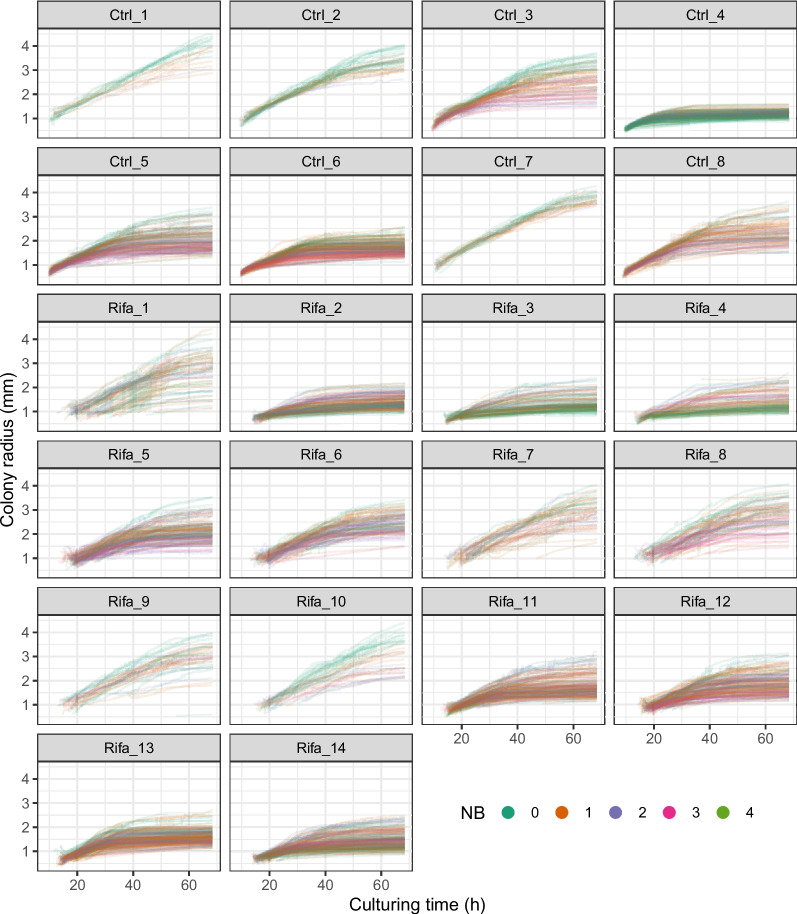
Growth curves of the colonies. Each curve describes the change in the radius size of a single colony (in Table 1, TN columns indicate their number). Their color indicates the number of close neighbors (NB) of the colony

The growth curves of colonies are shown in Fig. 3. Following the approach of Bärr et al. [11] we estimated the linear growth trend per culture for the first 24 h (Table 1). The average growth rate for Ctrl plates with less than 150 colonies was 60.3 $\mu m/h$ (SD: 5.6). Using a mixed effect model, colony growth rate estimates for Ctrl and Rifa groups are summarized in Table 1.

**Table 1:** Hourly growth rate of the bacterial colony radius during the first 24 h. Table A shows values estimated by linear regression in each culture. The plate identifiers, taken from the name of the library of digital images hosted on Figshare by Bärr et al. [11] are the number of colonies tracked in the TN column, the ID column is the culture identifier used in the paper of Bärr et al. [11] and the N column is the number of colonies per culture. Table B shows the estimates of the radius growth within treatment groups using mixed-effect linear regression. Column NB indicates an additional grouping variable, the numbers indicate the number of close neighbors of colonies. The row without an NB value shows the estimates for all colonies

Plate	TN	Estimate	SE	Bärr et al. [11]		Group	TN	NB	All plates		TN	Plates with $N<150$	
		($\mu m/h$)		ID	N				Estimate	SE		Estimate	SE
									($\mu m/h$)			($\mu m/h$)	
A)						B)							
Ctrl_1	16	55.3	0.84	21	16	Ctrl	749		51.0	0.14	235	58.7	0.22
Ctrl_2	31	66.3	0.51	22	31		233	0	53.1	0.28	66	65.0	0.42
Ctrl_3	76	56.8	0.45	20	78		277	1	52.9	0.23	99	58.9	0.34
Ctrl_4	200	36.4	0.28	19	1,186		177	2	48.2	0.23	52	53.0	0.40
Ctrl_5	134	50.6	0.29	18	162		55	3	43.2	0.35	15	51.4	0.65
Ctrl_6	180	44.2	0.25	15	275		7	4	48.2	0.75	3	55.6	0.77
Ctrl_7	22	66.6	0.71	16	23								
Ctrl_8	90	56.7	0.33	17	102								
Rifa_1	54	16.3	2.52	5	71	Rifa	1757		36.5	0.29	321	33.9	0.79
Rifa_2	214	33.8	0.70	6	925		490	0	35.2	0.65	87	37.3	1.60
Rifa_3	165	42.4	0.73	7	1,200		665	1	38.6	0.43	119	42.4	1.31
Rifa_4	153	41.2	0.95	8	1,509		432	2	37.0	0.55	80	28.8	1.50
Rifa_5	136	23.2	1.45	10	158		139	3	36.1	0.76	31	31.0	1.82
Rifa_6	91	31.6	1.29	9	93		31	4	35.4	1.66	4	26.3	5.76
Rifa_7	43	37.6	2.30	11	44								
Rifa_8	68	34.3	1.86	12	76								
Rifa_9	36	40.4	2.15	13	40								
Rifa_10	29	46.8	2.20	14	33								
Rifa_11	181	40.6	0.67	1	288								
Rifa_12	166	29.3	1.01	2	207								
Rifa_13	206	44.4	0.59	3	425								
Rifa_14	215	33.1	0.61	4	662								

## Discussion

The best model detected all colonies in the figure in 0.31 seconds, regardless of the number of colonies. Bärr et al. [11] provided partial detection times for 16 cultures in their Supplementary Table 3, giving a mean per image of 8.8 s (SD: 7.35 s), which is 28 times our CNN result.

The median time to detect the first colonies in the Ctrl group (9.4 h) was 0.4 h later than that reported by Bärr et al. [11] (9 h). In the Rifa group, however, instead of 17.4 h [11], we obtained 13.2 h as the median of the appearance times. Thus, instead of 8.4 h [11], we obtained a 3.8 h difference in the median of the appearance times of the first colonies in the two groups.

The hourly colony growth rates for the first 24 h in Ctrl group with less than 150 colonies were estimated by Bärr et al. [11] to be 60.4 $\mu m$. Using the same statistical approach, our trained CNN estimated 60.3 $\mu m$, a difference of only 0.1 $\mu m$ (0.17%). However, due to repeated measurements, we believe that a more correct approach is to use the mixed-effect model, which yields 58.7 $\mu m$ for the same subset, with a 1.7 $\mu m$ difference (2.8%) from the reference. For estimates that do not consider the number of neighbors, we can see that the rate for cultures with less than 150 colonies is always higher than the rate calculated from the sum of all cultures. This is more substantial in the Ctrl group (7.7 $\mu m/h$) and less in the Rifa group (2.6 $\mu m/h$). In both approaches, the Ctrl group shows that the growth rate decreases with the increasing number of neighbors up to the subgroup with 3 neighbors. Those with 4 neighbors show an increased growth rate, however, as Table 1 shows, the number of colonies with 4 neighbors is very low, therefore, the estimates for these are not really reliable. In the analyses using all Rifa cultures, we see that colonies without close neighbors grow at a lower rate than those with close neighbors, among which the increase in the number of close neighbors indicates a clear decrease in rate. No such regularity is seen in the Rifa cultures, with less than 150 colonies. Comparing Fig. 3 showing the growth curves of colonies with the Supplementary Fig. 10 of Bärr et al. [11] we see that the final colony sizes of our estimates exceed in several plates the values presented by Bärr et al. [11] for the same ones. A visual inspection of the different cultures indicates that while the predicted bounding boxes are narrower in the case of the smaller colonies, more closely approximating the boundaries of the colonies, they can deviate significantly in the large colonies. If the aforementioned minimal deviation in growth rates in the first 24 hours is reconsidered in this light, it can be explained by the fact that until the end of that period, the colonies are still quite small, and the predictions do not distort the size of the bounding box. We believe that the more inaccurate bounding box estimation of large colonies may be because the images used in the training set were taken from cultures that were incubated for 24-48 hours. As a consequence, only a few colonies could have grown as large as on the 68-hour cultures of Bärr et al. [11]. This imprecision could be reduced by using a training set that includes longer incubation time with larger colonies.

Based on our results, we believe that CNN-based bacterial colony detection and bacterial colony growth dynamics analyses could become an effective tool for bacteriological work and research.

## Data Availability

The data used in the study is freely available at https://doi.org/10.6084/m9.figshare.22022540.v3 [12] and https://doi.org/10.6084/m9.figshare.12951152.v1 [11]. The codes used during the current study are available from the corresponding author upon reasonable request.

## References

[1] Anderson J, Eftekhar F, Aird M, Hammond J (1979). Role of bacterial growth rates in the epidemiology and pathogenesis of urinary infections in women. J Clin Microbiol..

[2] Bourchookarn A, Paddock C, Macaluso K, Bourchookarn W (2022). Association between growth rate and pathogenicity of spotted fever group Rickettsia. J Pure Appl Microbiol.

[3] McMeekin T (1997). Quantitative microbiology: a basis for food safety. Emerg Infect Dis.

[4] Madrid RE, Felice CJ, Valentinuzzi ME (1999). Automatic on-line analyser of microbial growth using simultaneous measurements of impedance and turbidity. Med Biol Eng Comput.

[5] Lindqvist R (2006). Estimation of staphylococcus aureus growth parameters from turbidity data: characterization of strain variation and comparison of methods. Appl Environ Microbiol.

[6] Fisher RA, Gollan B, Helaine S (2017). Persistent bacterial infections and persister cells. Nat Rev Microbiol..

[7] Levin-Reisman I (2010). Automated imaging with scanlag reveals previously undetectable bacterial growth phenotypes. Nat Methods..

[8] Levin-Reisman I, Fridman O, Balaban NQ (2014). Scanlag: high-throughput quantification of colony growth and lag time.

[9] Barr D (2016). Serial image analysis of mycobacterium tuberculosis colony growth reveals a persistent subpopulation in sputum during treatment of pulmonary tb. Tuberculosis..

[10] Vulin C, Leimer N, Huemer M, Ackermann M, Zinkernagel AS (2018). Prolonged bacterial lag time results in small colony variants that represent a sub-population of persisters. Nat Commun.

[11] Bar J, Boumasmoud M, Kouyos RD, Zinkernagel AS, Vulin C. Efficient microbial colony growth dynamics quantification with ColTapp, an automated image analysis application. Sci Rep 2020;10:16084. 10.1038/s41598-020-72979-4. 10.1038/s41598-020-72979-4 PMC7528005 32999342

[12] Makrai L (2023). Annotated dataset for deep-learning-based bacterial colony detection. Sci Data..

[13] Balmages I (2023). Use of the speckle imaging sub-pixel correlation analysis in revealing a mechanism of microbial colony growth. Sci Rep.

[14] Majchrowska S, et al. Agar a microbial colony dataset for deep learning detection. arXiv preprint arXiv:2108.01234. 2021.

[15] Pawłowski J, Majchrowska S, Golan T (2022). Generation of microbial colonies dataset with deep learning style transfer. Sci Rep..

[16] Wu Y, Kirillov A, Massa F, Lo W-Y, Girshick R. Detectron2. 2019. https://github.com/facebookresearch/detectron2. Accessed 6 June 2023.

[17] Ren S, He K, Girshick R, Sun J. Faster R-CNN: Towards real-time object detection with region proposal networks. In: Proceedings of the 28th International Conference on Neural Information Processing Systems - Volume 1, NIPS’15, 91–99. Cambridge: MIT Press; 2015.

[18] R Core Team. R: A Language and Environment for Statistical Computing. Vienna: R Foundation for Statistical Computing; 2022.

[19] Robinson D, Hayes A, Couch S. broom: Convert Statistical Objects into Tidy Tibbles. R package version 1.0.4. 2023. https://github.com/tidymodels/broom. Accessed 6 June 2023.

[20] Bolker B, Robinson D. broom.mixed: Tidying Methods for Mixed Models. R package version 0.2.9.4. 2022. https://github.com/bbolker/broom.mixed. Accessed 6 June 2023.

[21] Wickham H (2016). ggplot2: Elegant Graphics for Data Analysis.

[22] Pebesma E. Simple Features for R: Standardized Support for Spatial Vector Data. R J 10:439–446. 2018. 10.32614/RJ-2018-009.

[23] Tennekes M (2018). tmap: Thematic maps in R. J Stat Softw.

[24] Dahl DB, Scott D, Roosen C, Magnusson A, Swinton J. xtable: Export Tables to LaTeX or HTML. R package version 1.8-4. 2019. https://cran.r-project.org/web/packages/xtable. Accessed 6 June 2023.

